# Sex-Specific Regulation of miR-29b in the Myocardium Under Pressure Overload is Associated with Differential Molecular, Structural and Functional Remodeling Patterns in Mice and Patients with Aortic Stenosis

**DOI:** 10.3390/cells9040833

**Published:** 2020-03-30

**Authors:** Raquel García, Ana B. Salido-Medina, Aritz Gil, David Merino, Jenny Gómez, Ana V. Villar, Francisco González-Vílchez, María A. Hurlé, J. Francisco Nistal

**Affiliations:** 1 Department of Physiology and Pharmacology, School of Medicine, University of Cantabria, 39011 Santander, Spain; villarav@unican.es; 2 Instituto de Investigación Marqués de Valdecilla (IDIVAL), 39011 Santander, Spain; anabelensalidomedina@gmail.com (A.B.S.-M.); aritzgil89@hotmail.com (A.G.); david.merino@hotmail.com (D.M.); jennygomezd@gmail.com (J.G.); cargvf@gmail.com (F.G.-V.); 3 Service of Cardiology, Hospital Universitario Marqués de Valdecilla, 39008 Santander, Spain; 4 Instituto de Biomedicina y Biotecnología de Cantabria (IBBTEC), CSIC-Universidad de Cantabria, 39011 Santander, Spain; 5 Service of Cardiovascular Surgery. Hospital Universitario Marqués de Valdecilla, 39008 Santander, Spain; 6 Department of Medical and Surgical Sciences, School of Medicine, University of Cantabria, 39011 Santander, Spain; 7 Centro de Investigación Biomédica en Red Cardiovascular (CIBERCV), Instituto de Salud Carlos III, 28029 Madrid, Spain

**Keywords:** miR-29b, aortic stenosis patients, pressure overload, cardiac remodeling, sex differences

## Abstract

Pressure overload in patients with aortic stenosis (AS) induces an adverse remodeling of the left ventricle (LV) in a sex-specific manner. We assessed whether a sex-specific miR-29b dysregulation underlies this sex-biased remodeling pattern, as has been described in liver fibrosis. We studied mice with transverse aortic constriction (TAC) and patients with AS. miR-29b was determined in the LV (mice, patients) and plasma (patients). Expression of remodeling-related markers and histological fibrosis were determined in mouse LV. Echocardiographic morpho-functional parameters were evaluated at baseline and post-TAC in mice, and preoperatively and 1 year after aortic valve replacement (AVR) in patients with AS. In mice, miR-29b LV regulation was opposite in TAC-males (down-regulation) and TAC-females (up-regulation). The subsequent changes in miR-29b targets (collagens and GSK-3β) revealed a remodeling pattern that was more fibrotic in males but more hypertrophic in females. Both systolic and diastolic cardiac functions deteriorated more in TAC-females, thus suggesting a detrimental role of miR-29b in females, but was protective in the LV under pressure overload in males. Clinically, miR-29b in controls and patients with AS reproduced most of the sexually dimorphic features observed in mice. In women with AS, the preoperative plasma expression of miR-29b paralleled the severity of hypertrophy and was a significant negative predictor of reverse remodeling after AVR; therefore, it may have potential value as a prognostic biomarker.

## 1. Introduction

Degenerative stenosis of the aortic valve (AS) is the heart valve disorder most frequently requiring surgery in adult patients [1]. This is a disease affecting elderly people, and more than 70% of patients are age 70 or above. The degenerative process progressively alters the biomechanical behavior of the aortic leaflets and root during long time periods, starting with valve sclerosis and finally producing a severe restriction in the systolic excursion of the cusps that impedes outflow [2,3]. This, in turn, generates a sustained, progressive pressure overload to the left ventricle (LV), thus triggering hypertrophic remodeling of cardiomyocytes to compensate for the elevation in mechanical wall stress [3].

The remodeling response also encompasses adaptive changes in the extracellular matrix (ECM), including an increased deposition of fibrillar collagens, to provide a structural scaffold and signaling control to cardiac cells, thereby preserving tissue integrity and cardiac function [4]. However, these plastic changes may become maladaptive over time, thus resulting in systolic and diastolic dysfunction, heart failure and eventually death [5,6].

Among the numerous neurohormonal factors and cellular mediators that orchestrate cardiac plasticity, microRNAs (miRNAs) have emerged as prominent players that hold promise for therapeutic interventions in cardiovascular disorders [7,8,9]. miRNAs are short segments of single-stranded non-coding RNA involved in the post-transcriptional regulation of gene expression. Mature miRNAs bind complementary sequences of target mRNAs and induce either translational repression or mRNA degradation [10].

The miR-29 family, comprising miR-29a, miR-29b, and miR-29c, has been well characterized to regulate many ECM-related genes, including almost all collagen isoforms [11,12,13,14]. miR-29 deregulation plays a role in establishing pathological feedback loops that sustain the progression of fibrosis in many tissues [11]. Repression of miR-29 by stressors releases their mRNA targets from posttranscriptional inhibition [12,13,14,15,16]. Accordingly, miR-29 has been reported to be down-regulated in patients and in rodent models of progressive fibrotic diseases including those of the heart, arteries, kidneys, lungs, liver, and skin, in which functional failure of the affected organ is the usual outcome [11,15,17,18,19,20,21,22,23,24,25]. 

Gain- or loss-of-function studies using genetic or pharmacological tools in mice have examined the roles of mir-29 during cardiac remodeling induced by stressors, such as myocardial infarction [12], angiotensin II infusion [23,24] or pressure overload [14,26]. However, the results of these studies are contradictory: miR-29 has been found to be both protective [12,14,23,24] and harmful [26] in cardiac fibrosis and/or hypertrophy, even under similar experimental conditions [14,26]. The mechanism underlying these inconsistencies has not been elucidated.

The existence of sex-dependent patterns of LV remodeling under pressure overload has long been recognized [27,28]. These differences are multidimensional and include various aspects of remodeling, including tissue composition at the molecular and cellular levels, chamber geometry, systolic and diastolic pump function, or metabolics [29,30,31,32,33]. The complexities of these differential sex patterns are partially understood but remain mostly unclear.

Sexually dimorphic regulation of miR-29 underlies the sex-related differences in the susceptibility of mice to liver fibrosis during carbon tetrachloride (CCl4) treatment. Estrogens confer resistance against liver fibrosis in females by inducing the expression of miR-29 and thus reducing collagen deposition [21]. In contrast, down-regulation of miR-29 in males is correlated with the early development of liver fibrosis [20,21]. 

We hypothesized that this sex-specific regulation of miR-29 and its targeted mRNAs may also occur in the myocardium under pressure overload stress. Herein, we provide the first demonstration that sex differences in LV remodeling (molecular, structural and morpho-functional) involve sex-specific regulation of miR-29b in mice subjected to TAC and in the clinical aortic stenosis setting. 

A better understanding of the influence of sex on the molecular mechanisms underlying maladaptive myocardial remodeling may facilitate the development of personalized, sex-specific treatment strategies for pressure overload hypertrophy. 

## 2. Materials and Methods

### 2.1. Pressure Overload Studies in Mice

The study was approved by the University of Cantabria Institutional Laboratory Animal Care and Use Committee (reference IP0415) and was conducted following the guidelines of directive 2010/63/EU of the European Parliament. All animals received humane care, and every effort was made to minimize suffering. We randomized 14–16-week-old male and female C57BL/6 mice to either sham operation or constriction of the aortic arch (TAC). Mice were anesthetized with an intraperitoneal injection of ketamine (100 mg/kg) and xylazine (5 mg/kg) and subjected to TAC with Nistal’s double loop-clip technique [34]. Briefly, before the operation, a suture (7/0 polypropylene) was prepared with two knots separated by a distance calculated with an algorithm incorporating the echocardiographically measured arch diameter of the animal and the intended degree of constriction. The surgery was performed under spontaneous ventilation with an extrapleural approach via a manubrium sternotomy. The aorta was encircled twice with the suture, and the loop was closed with a vascular microclip under both knots. After a 4-week follow-up, the mice were euthanized, and the LV samples were snap-frozen in liquid nitrogen or fixed in 4% paraformaldehyde for histology.

#### 2.1.1. Echocardiography

Transthoracic echocardiography was performed in mice under sedation with isoflurane (2.5%) with ultrasound equipment (Vevo-770, Fujifilm-VisualSonics, Toronto, ON, Canada) with a high-resolution transducer centered at 30 MHz. The operator was blinded to the study groups. Trans-coarctation pressure gradients were measured with pulsed-wave Doppler analysis at the distal arch. LV end-diastolic (LVEDd) and end-systolic (LVESd) internal diameters, interventricular septum thickness (IVST), and LV posterior wall thickness (PWT) were measured according to the recommendations of the American Society of Echocardiography. The degree of geometric concentricity of the LV was assessed with the relative PWT (rPWT) calculated as rPWT = (2*PWT/(LVEDd). Cardiac mass was estimated with Devereux’s formula and indexed to body weight. The mitral annular plane systolic excursion (MAPSE) measurements were obtained from four-chamber views with M-mode imaging. The LV ejection fraction (LVEF) and MAPSE were used as surrogates for the short-axis and longitudinal systolic functions, respectively. Parameters of diastolic LV function (E/e′) were obtained with pulsed-wave mitral inflow analysis, and tissue Doppler imaging to obtain the ratio of peak early transmitral flow velocity (E) to peak early myocardial tissue velocity (e′).

#### 2.1.2. Gonadectomy

Bilateral orchiectomy (ORCH) or ovariectomy (OVX) were conducted under anesthesia with ketamine (100 mg/kg) and xylazine (5 mg/kg). To avoid any residual hormonal effects, two months were allowed between gonadectomy and TAC or sham surgery. Plasma levels of testosterone and estradiol were measured by immunoassay with the ADVIA Centaur Immunoassay Systems (Siemens Healthineers, Erlangen, Germany).

### 2.2. Studies in Cardiac Fibroblasts

Adult cardiac fibroblasts were isolated from 2-month-old C57BL6 male and female mice through enzymatic digestion, as previously described [35]. For experimental procedures, low passage cells (p2) were seeded onto 35 mm cell culture dishes and incubated at 37 °C, in 5% CO_2_, for 15–20 h. Cell growth was arrested by incubation in DMEM containing 0.5% DBS for 12 h. Cells were incubated in serum-free medium with 17β-estradiol (5 and 10 nM) or TGF-β1 (10 ng/mL) for 24 h. Total RNA was obtained and reverse transcribed to perform qPCR.

### 2.3. Pressure Overload Studies in Patients

The study followed the Declaration of Helsinki guidelines for investigations in human subjects. The Institutional Ethics and Clinical Research Committee of Cantabria approved the study, and all patients provided written informed consent. The study was performed with LV myocardial intraoperative biopsies and plasma samples obtained from a cohort of 103 patients (52 men and 51 women) with isolated severe AS and undergoing aortic valve replacement surgery at the University Hospital Marqués de Valdecilla in Santander, Spain. Patients with more than mild aortic or mitral regurgitation or with major coronary stenosis >50%, previous cardiac operations, malignancies, or poor renal or hepatic function were deemed ineligible for the study. The control group was a cohort of 40 surgical patients (17 men and 23 women) with pathologies (atrial septal defect: *n* = 24, aortic aneurysm: *n* = 10, mitral stenosis: *n* = 3, left atrial myxoma: *n* = 2, pulmonary valve fibroelastoma: *n* = 1) that did not include pressure or volume overload, coronary heart disease or cardiomyopathies. Subepicardial biopsies (40 mg) were taken from the LV lateral wall with a Tru-cut needle during the surgical procedure. The same surgeon harvested all samples in a protocolized manner and always collected from the same location in the margo obtusus of the heart. Peripheral venous blood samples were collected from control group and patients with AS; in both groups, the samples were drawn 24 h preoperatively. To minimize platelet degranulation, the blood was drawn from an antecubital vein without a tourniquet, with a syringe with a wide-gauge needle, and was then gently transferred to a collection tube containing EDTA. Within 30 min of collection, the plasma was separated by centrifugation at 1000× *g* for 30 min, and aliquots were stored at −80 °C until analysis.

#### Echocardiography

A two-dimensional transthoracic echocardiogram was performed (Philips-Hewlett Packard, IE 33, Amsterdam, Netherlands) preoperatively and 1 year after surgery. Images were digitalized and analyzed off-line (Xcelera software, Philips, Amsterdam, Netherlands). LVEDd, LVESd, IVST, and PWT were measured according to the American Society of Echocardiography guidelines with bidimensional or M-mode images depending on the quality and angulation between the ultrasound beam and the LV. The rPWT was calculated. LVEF was calculated with the Quiñones formula, and LVM was estimated according to the Devereux formula and indexed to patient height in meters to the 2.7th power.

### 2.4. Determination of mRNA and miRNA Expression by q-PCR

Total RNA was obtained from tissue and cell cultures with TRIzol (Invitrogen, Carlsbad, CA, USA) extraction. Real-time PCR was conducted with specific TaqMan assays (Thermo Fisher Scientific, Waltham, MA, USA) for the following genes: collagen 1α1 (Col 1α1), collagen 3α1 (Col 3α1), glycogen synthase kinase 3 β (GSK-3β), and miR-29b. The housekeeping genes were 18S and RNU6b. Transcripts were determined in triplicate in two different assays.

Total RNA was isolated from plasma samples with a miRNeasy serum/plasma kit (Qiagen, Hilden, Germany). The isolation efficiency of the plasma miRNAs was assessed with spiked-in *Caenorhabditis elegans* miRNA (cel-miR-39) (Qiagen, Hilden, Germany) lacking sequence homology to human miRNAs, as described previously [36]. The oligonucleotides were spiked into the samples during RNA isolation after incubation of the plasma with the denaturing solution. The plasma miRNAs were reverse transcribed with specific primers for miR-29b and cel-miR-39 (Thermo Fisher Scientific, Waltham, MA, USA), and the plasma miR-29b levels were normalized to those of cel-miR-39. To ensure that the isolation efficiency was homogeneous among the samples, we repeated the extraction procedure, if necessary, until the cel-miR-39 cycle threshold fell within a range of 23.0 ± 1.0.

### 2.5. Histology

Hearts were fixed in paraformaldehyde (3.7% in PBS) for 48 h and embedded in paraffin. Four short-axis sections at the level of the papillary muscles (*n* = 4 mice per experimental condition) were stained with Masson’s trichrome. With this technique, muscle fibers are stained red, collagens are stained blue, the cytoplasm is stained light red or pink, and cell nuclei are stained dark brown to black. Digital photographs of the LV sections were captured with a camera (Axiocam MRc5, Zeiss, Oberkochen, Germany) attached to a Zeiss Axioplan microscope. The fractional area of fibrosis was determined (ImageJ software), and the results are expressed as the percentage of the total LV myocardial area stained in blue. The operator was blinded to the experimental groups.

### 2.6. Western Blotting

Thirty micrograms of protein lysates were electrophoresed on a 10% sodium dodecyl sulfate–polyacrylamide gel and transferred onto a polyvinylidene difluoride membrane (Bio-Rad, Hércules, CA, USA). The primary antibodies were goat polyclonal antibody to GSK-3α/β (Cell Signaling Technology, Danvers, MA, USA) and rabbit polyclonal antibody to GAPDH (Santa Cruz, Dallas, TX USA). After incubation with the appropriate secondary antibodies, proteins were immunodetected with infrared fluorescence (Odyssey Imager LI-COR Biosciences, Lincoln, NE, USA). The results are expressed as the optical density of the samples normalized to that of GAPDH. Samples from three mice per group were tested in two independent experiments.

### 2.7. Statistics

Data were assessed for normality with the Kolmogorov–Smirnov test. Values are reported as means ± SEM. Continuous variables from multiple groups were compared with one way ANOVA or Kruskal-Wallis test. The influence of sex and pressure overload on gene and protein expression was assessed with two-way ANOVA and the influence on echocardiographic parameters was assessed with repeated-measures two-way ANOVA. Bonferroni or Dunn post-hoc tests were used when appropriate. Correlations between miR-29b mRNA levels and echocardiographic parameters were performed with Pearson’s correlation analysis. A comparison of correlations was performed with cocor [37]. Logistic regression analysis was used to identify predictors of LV mass normalization 1 year after aortic valve replacement in patients with AS. The Hosmer–Lemeshow test was used to evaluate the goodness of fit of the model. Post-hoc assessment of the regression model in patients was performed with the bootstrapping method with 1000 iterations. The receiver operating characteristic (ROC) curve was calculated to assess the capability of the model to discriminate patients with normalized LVM 1 year after AVR from those maintaining residual hypertrophy. Significance levels were as follows: * *p* < 0.05, ** *p* < 0.01, *** *p* < 0.001. Statistical packages: GraphPad Prism 5.03, PASW Statistics 22 (SPSS Inc., Chicago, IL, USA) and R software.

## 3. Results

### 3.1. The Myocardial Expression of miR-29b and its Regulation Under Pressure Overload Exhibit Sexual Dimorphism in Mice: Roles of Gonadal Hormones

LV pressure overload was induced in male and female mice by TAC for 4 weeks (4wk-TAC) with Nistal’s technique [34]. This customized procedure, in contrast to the standard Rockman method [38], ensures identical levels of LV pressure overload regardless of somatometric differences between males and females. At 4 weeks after TAC or sham surgery, miR-29b expression was determined in the LV myocardium.

**Figure 1:**
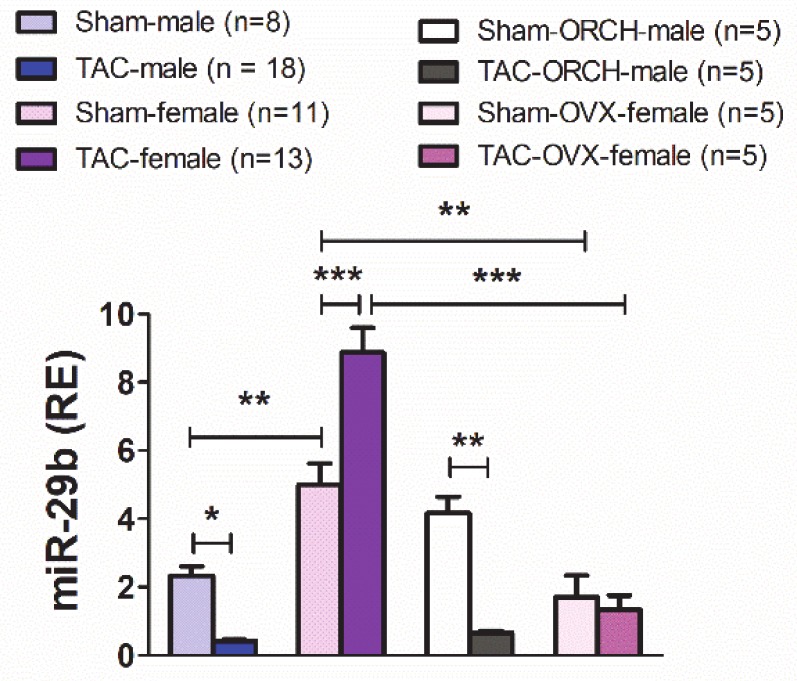
Relative expression (RE vs. RNU6b) of miR-29b in the LV from male, female, orchiectomized (ORCH) male and ovariectomized (OVX) female mice subjected to a 4-week transverse aortic constriction (TAC) or sham (no-TAC) surgery. Data are expressed as mean ± SEM. * *p* < 0.05, ** *p* < 0.01, *** *p* < 0.001 (two-way ANOVA followed by Bonferroni post hoc test).

Under control conditions (Figure 1), sham-females exhibited significantly higher levels of miR-29b in the LV than sham-males. Unexpectedly, TAC induced myocardial miR-29b up-regulation in 4wk-TAC-females but down-regulation in 4wk-TAC-males. This opposite regulation of miR-29b expression between sexes took place under identical LV pressure overload, as indicated by the trans-coarctation gradients (TAC-male: 58.0 ± 2.5 mm Hg; TAC-female: 54.4 ± 2.1 mm Hg; Supplemental Figure S1).

We next sought to investigate the contribution of gonadal hormones to the sexually dimorphic myocardial expression of miR-29b, under both normal afterload (sham-mice) and pressure overload (TAC-mice). To this end, we subjected a series of female and male mice to ovariectomy (OVX) or orchiectomy (ORCH) two months before TAC or sham (no-TAC) surgery. The testosterone or 17β-estradiol circulating levels were below the detection limits two months after castration.

The trans-coarctation pressure values of TAC-OVX-females and TAC-ORCH-males are shown in Figure S1. As shown in Figure 1, the LV expression levels of mir-29b were significantly lower in sham- and TAC-OVX-females than in non-castrated sham- or TAC-females, respectively. In OVX-females, TAC did not induce significant changes in the LV levels of miR-29b. In sham-males, castration did not produce significant changes in the LV expression of miR-29b. Pressure overload-induced a quantitatively similar down-regulation of miR-29b in both TAC-intact-males and TAC-ORCH-males (Figure 1). These results indicate that the sexually dimorphic expression of miR-29b in the LV, under both physiological afterload conditions and pressure overload, is dependent on gonadal estrogens but unrelated to gonadal androgens.

### 3.2. The Regulation of miR-29b Expression in Primary Cardiac Fibroblasts by the Profibrotic Cytokine TGF-β Exhibits Sexual Dimorphism

**Figure 2:**
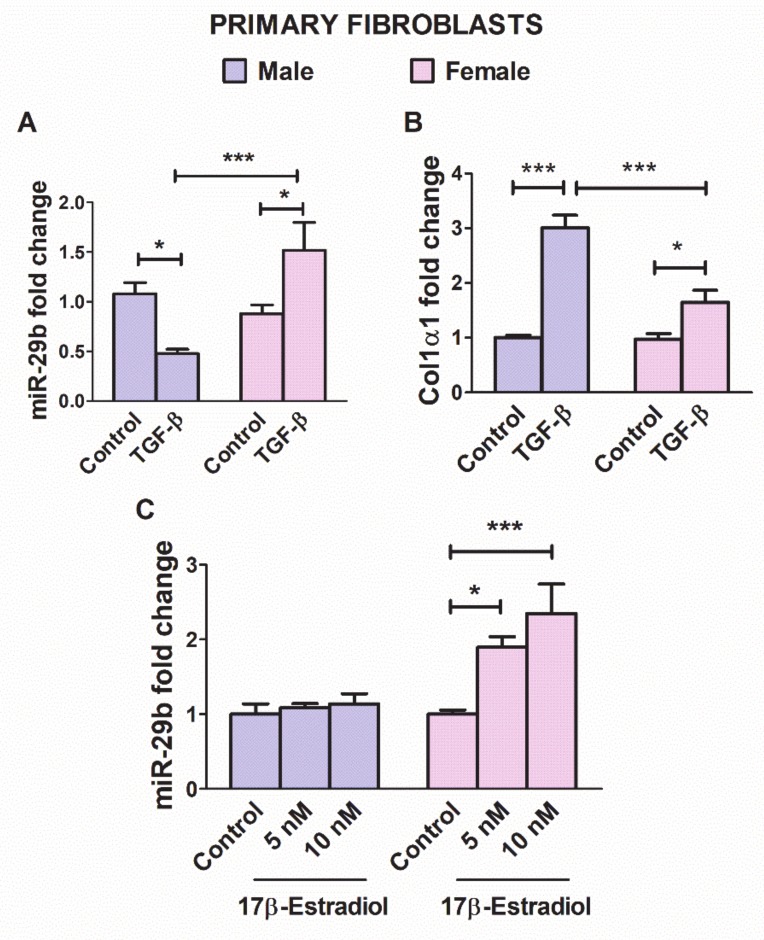
Relative expression changes in miR-29b (**A**) and Col1α1 (**B**) in cultured cardiac fibroblasts isolated from male and female myocardium, stimulated with TGF-β1 (10 ng/mL) for 24 h in serum-free medium. Relative changes in the expression of miR-29b in cardiac fibroblasts from male and female mice stimulated with 17β-estradiol (5 and 10 nM) for 24 h (**C**). Data are reported as mean ± SEM * *p* < 0.05, *** *p* < 0.001 (two way ANOVA and Bonferroni’s post-hoc test).

TGF-β is a crucial player in the myocardial remodeling response under pressure overload [29,35,39]. Down-regulation of miR-29b in fibroblasts is among the mechanisms underlying the fibrotic effect of TGF-β in the stressed LV myocardium [12,14,24]. The miR-29b expression is decreased by TFG-β, thereby inducing fibrosis [19,24,25]. Therefore, we next sought to assess whether miR-29b regulation by TGF-β1 in cultured primary cardiac fibroblasts might differ depending on the sex of the animal from which the cells were obtained. As expected, the expression of miR-29b in cells exposed to TGF-β1 (10 ng/mL) for 24 h was significantly down-regulated in fibroblasts from males but up-regulated in fibroblasts from females (Figure 2A). Moreover, under TGF-β stimulation, male fibroblasts exhibited higher Col1α1 expression levels than female fibroblasts, thus indicating sexually dimorphic responses to TGF-β1 stimulation in cardiac cells (Figure 2B).

The miR-29b expression has been reported to be modulated by estradiol in a hepatic cell line [21]. Therefore, we assessed whether the expression of mir-29b in primary LV cardiac fibroblasts was estradiol sensitive. As shown in Figure 2C, miR-29b was upregulated by estradiol in a concentration-dependent manner in fibroblasts from female but not male mice.

### 3.3. The Morpho-Functional Echocardiographic Remodeling Exhibits Sexually Dimorphic Profiles

We assessed whether the sexually dimorphic regulation of miR-29b under pressure overload might influence the LV remodeling outcomes in males and females. We evaluated the morphological, geometric and functional changes in the LV 4 weeks after TAC, ensuring that the pressure overload to the LV was identical in all animals (Figure S1) regardless of body size [34].

**Figure 3:**
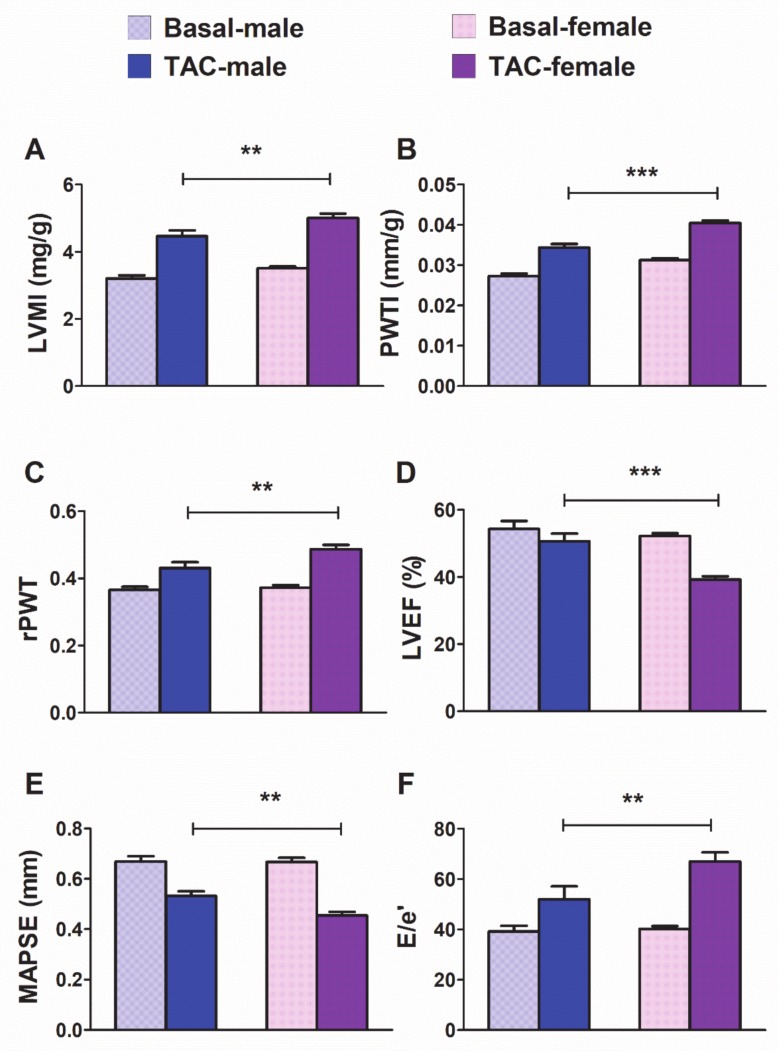
Morpho-functional echocardiographic changes induced by pressure overload in male (*n* = 19) and female (*n* = 29) mice subjected to 4-week transverse aortic constriction (TAC). LVMI: left ventricle (LV) mass indexed to body weight (**A**). PWTI: posterior wall thickness indexed to body weight (**B**). rPWT: relative posterior wall thickness (2xPWT/LVEDd) (**C**). LVEF: LV ejection fraction (**D**). MAPSE: mitral annular plane systolic excursion (**E**). E/e′: ratio of peak early transmitral flow velocity to peak early myocardial tissue velocity (e′) (**F**). Data are expressed as means ± SEM. ** *p* < 0.01; *** *p* < 0.001 (repeated-measures two-way ANOVA followed by Bonferroni test).

Pressure overload produced significant increases in hypertrophy-related echocardiographic parameters: LV mass index (LVMI) (Figure 3A) and posterior wall thickness index (PWTI) (Figure 3B) in males and females [LVMI (TAC: F_1,46_= 223, *p* < 0.001; sex: F_1,46_ = 8.8, *p* < 0.001); PWTI (TAC: F_1,46_ = 195.2 *p* < 0.001; sex: F_1,46_ = 43.9, *p* < 0.001)]. However, hypertrophy reached significantly higher values in 4wk-TAC-females than in 4wk-TAC-males. With regard to LV geometry, 4-wk-TAC induced an increase in the relative posterior wall thickness (a measure of LV concentricity) in both groups of mice (Figure 3C) [rPWT (TAC: F_1,46_ = 57,4, *p* < 0.001; sex: F_1,46_ = 6.8, *p* < 0.05)]. Notably, 4wk-TAC-females exhibited a more concentric pattern of remodeling than 4wk-TAC-males.

The systolic function in the short-axis (LVEF) (Figure 3D) was affected by pressure overload in a sex dependent manner [LVEF (interaction: F_1,46_ = 1.3, *p* < 0.01; TAC: F_1,46_ = 39.4, *p* < 0.001; sex: F_1,46_ = 15.4, *p* < 0.001)], and a greater reduction of the ejection fraction was observed in females than in males. The long-axis systolic function (Figure 3E) [MAPSE (TAC: F_1,46_ = 83.3, *p* < 0.001; sex: F_1,46_ = 6.4, *p* < 0.05)] was also depressed by TAC, and the reduction was greater in females than in males.

Regarding diastolic function (Figure 3F), pressure overload increased the E/e’ ratio (a proxy for LV filling pressure) in a sex dependent manner [E/e’ (interaction: F_1,22_ = 3.2, *p* < 0.05; TAC: F_1,22_ = 19.1, *p* < 0.001; sex: F_1,22_ = 2.5, *p* < 0.05)]. TAC increased E/e’ to a greater extent in females than in males.

Male and female results of morphological and functional echocardiographic parameters as percent change versus the basal values are represented in the Supplementary Materials (Figure S2).

### 3.4. The Correlation Between LV miR-29b Expression and Morpho-Functional Remodeling Exhibits Opposite Trends in Males and Females

**Figure 4:**
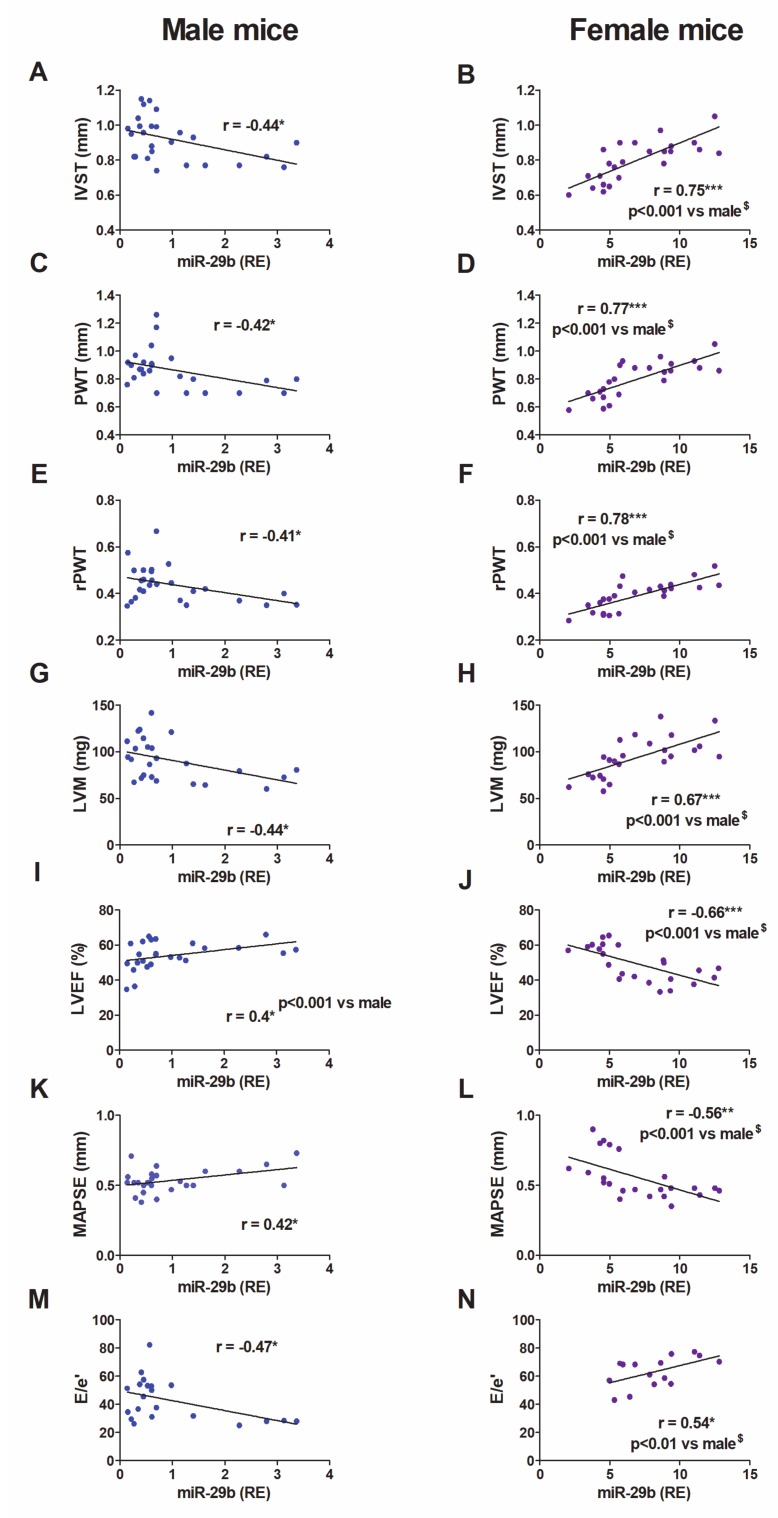
Relationship between LV miR-29b expression and morphological and functional echocardiographic parameters in mice. Linear regression and Pearson’s correlation analyses showing the relationship between LV miR-29b expression and morpho-functional echocardiographic parameters, in sham (*n* = 8 males; *n* = 11 females) and TAC (*n* = 17 males; *n* = 13 females) mice. IVS, interventricular septum (**A**,**B**); PWT, posterior wall thickness (**C**,**D**); rPWT, relative posterior wall thickness (**E**,**F**); LVM, LV mass (**G**,**H**); LVEF, LV ejection fraction (**I**,**J**); MAPSE, mitral annular plane systolic excursion (**K**,**L**); E/e′, the ratio of peak early transmitral flow velocity (E) to peak early myocardial tissue 1 velocity (e′) (**M**,**N**), r, Pearson’s correlation coefficient. * *p* < 0.05, ** *p* < 0.01, *** *p* < 0.001. **^$^** Comparative analysis of female vs. male correlations with cocor.

We sought to determine the relationship between LV miR-29b expression and sex-dependent patterns of morpho-functional remodeling (Figure 4). In male mice, the expression levels of miR-29b in the LV exhibited an inverse relationship with LV hypertrophy (IVS, PWT, and LVM) and concentricity (rPWT) (Figure 4A,C,E,G). Moreover, male mice with the highest LV expression of miR-29b displayed the best systolic function in the short-axis (LVEF) (Figure 4I) and long-axis (MAPSE) (Figure 4K), and the lowest LV filling pressures (E/e’) (Figure 4M). These results strongly support the protective role of miR-29b against myocardial remodeling in male mice during pressure overload.

Unexpectedly, the relationships were in the opposite direction in female mice (Figure 4B–N): female mice with the highest LV expression levels of miR-29b exhibited the highest values of hypertrophy (LVM, PWT, and IVS) and concentricity (rPWT), the worst systolic function in both the short-axis (LVEF) and long-axis (MAPSE), and the most severe diastolic dysfunction (E/e’). These results strongly suggest that the effect of miR-29b in females, rather than being protective, is harmful to the myocardium under pressure overload.

### 3.5. The Regulation by Pressure Overload of Relevant LV Remodeling Related Elements Exhibits a Sexually Dimorphic Pattern in Mice

In TAC-mice, we assessed the changes in the expression of several remodeling-related genes that are activated under pressure overload and induce pathological fibrosis and hypertrophy of the myocardium [6].

**Figure 5:**
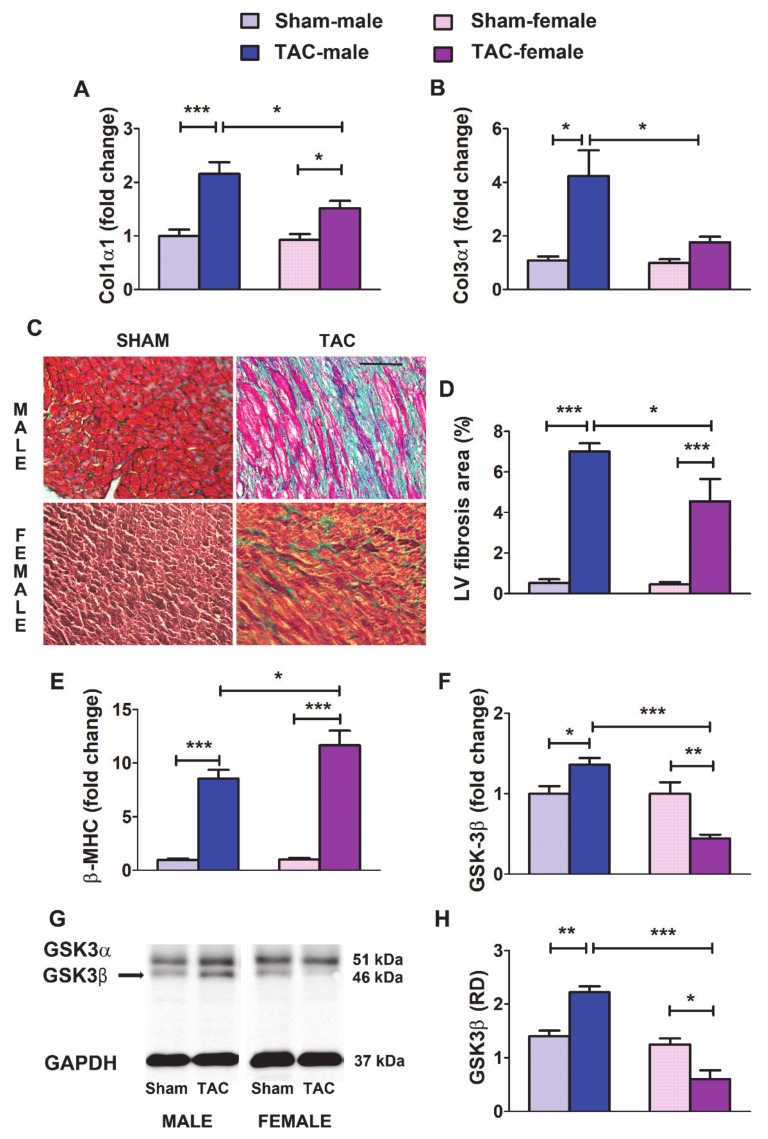
Regulation by pressure overload of relevant LV remodeling related elements in mice. **A** and **B**: Fold change in mRNA levels of Col1α1 (**A**) and Col1α3 (**B**) in the LV from male and female mice subjected to sham surgery or transverse aortic constriction (TAC) for 4 weeks (sham males, *n* = 9; sham females, *n* = 11; TAC males, *n* = 15; TAC females, *n* = 12). (**C**) Representative images of LV sections showing myocardium stained with Masson’s trichrome. With this technique, muscle fibers are stained red, fibrosis is stained blue, the cytoplasm is stained light red or pink, and cell nuclei are stained dark brown to black. (**D**) Average fractional LV area of fibrosis in 4 sections from three to six mice per group. (**E,F**) Fold change in mRNA levels of β-MHC (**E**) and GSK-3β (**F**) in the LV from sham and TAC mice of both sexes. (**G**) Representative western blot images showing the protein levels of GSK-3β in the LV. (**H**) Average relative optical density (RD vs. GAPDH) quantified in three mice per group in two different experiments. Data are means ± SEM. * *p* < 0.05, ** *p* < 0.01; *** *p* < 0.001 (ANOVA followed by the Bonferroni post-hoc test).

Fibrillar collagen types I and III, the predominant components of the cardiac ECM, are validated targets of miR-29b [12,14]. Herein, the expression levels of transcripts encoding Col1α1 (Figure 5A) and Col3α1 (Figure 5B) were up-regulated in the LV from 4-wk-TAC-males and females. However, 4wk-TAC-females, in line with their higher expression of miR-29b, exhibited significantly lower levels of both ECM elements than 4wk-TAC-males (Figure 5A,B). Accordingly, the percentage of the histological area stained for fibrosis was higher in the LV in males than in females (Figure 5C,D).

The expression of the sarcomeric protein β-MHC is an early and sensitive marker of cardiac hypertrophy in the myocardium under overload stress in rodents [40]. As shown in Figure 5E, β-MHC was significantly up-regulated after TAC, in both males and females. However, β-MHC rose to higher levels in 4wk-TAC-females, a result consistent with the more severe hypertrophy in females. 

The glycogen synthase kinase GSK-3β is a negative regulator of cardiomyocyte growth whose repression is required for the myocardial hypertrophic response under pressure overload [41]. Targeting GSK-3β contributes to the pro-hypertrophic function of miR-29 in the pressure overloaded LV [26]. Herein, GSK-3β was significantly down-regulated, at both the mRNA and protein levels, in the LV from 4wk-TAC-females but up-regulated in 4wk-TAC-males (Figure 5F–H). The lower levels of GSK-3β in females than males may be associated with the more severe hypertrophy developed by females under pressure overload.

Overall, these results indicate that, in mice, the LV myocardium under identical pressure overload stress develops a remodeling response at 4 weeks, whose pattern is conditioned by sex: concentric hypertrophy prevails in females, whereas fibrosis dominates in males.

### 3.6. In Patients with Aortic Stenosis, the Morpho-Functional Remodeling of the LV Under Pressure Overload Exhibits Sex-Related Differences: Role of miR-29b

To evaluate the potential translation of our experimental data to humans, we determined the levels of miR-29b in the LV and plasma in patients of both sexes with LV pressure overload secondary to AS. Our results above revealed the estrogen dependence of miR-29b expression in the mouse LV and led us to assess whether the LV and plasma levels of miR-29b in humans might be affected by clinical and demographic characteristics such as age, sex and women’s hormonal status.

Because the age of our cohort of control women ranged from 28 to 83 years, we assessed whether the expression of miR-29b might be influenced by hormonal status in women free of pressure overload. When we analyzed the entire cohort of control patients of all ages (Figure S3), we observed that miR-29b expression levels in both the LV and plasma correlated inversely with age in control women (r = −0.42). However, this relationship was supported by the younger patients, and no relationship between age and miR-29b was found in the control women older than 50 years (r = 0.03). In control men, no relationship was evident between miR-29b and age.

**Figure 6:**
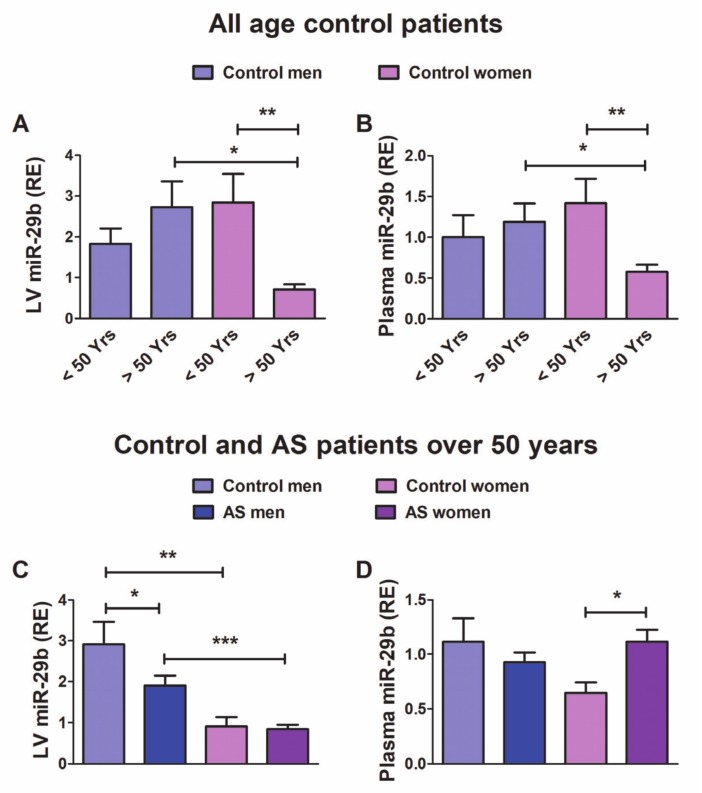
Expression levels of miR-29b in the LV tissue and plasma in patients. (**A**) relative miR-29b expression in the LV (RE vs. RNU6b) from control individuals under 50 years (*n* = 6 men, *n* = 8 women) and over 50 years (*n* = 11 men, *n* = 15 women) of age, and (**B**) in plasma (RE vs. Cel-39) from control individuals under 50 years (*n* = 5 men, *n* = 8 women) and over 50 years (*n* = 10 men, *n* = 13 women) of age. (**C**) miR-29b expression in the LV from control patients (11 men, 15 women) and patients with AS (45 men, 50 women) over 50 years of age, and (**D**) in plasma from control patients (10 men, 13 women) and patients with AS (38 men, and 46 women) over 50 years of age. Data are means ± SE. * *p* < 0.05, ** *p* < 0.01, *** *p* < 0.001 (Kruskal–Wallis followed by Dunn’s post hoc test).

We further assessed the differences in the expression levels of miR-29b between control individuals under 50 years (*n* = 6 men, *n* = 8 women) and over 50 years (*n* = 11 men, *n* = 15 women) of age. As shown in Figure 6A,B, postmenopausal (> 50 years) control women exhibited significantly lower expression of miR-29b in both the LV and plasma than did women under 50 years or age-matched men. In control men, we observed no difference among age groups.

These results indicate that the sexually dimorphic expression of miR-29b in the LV and plasma may be associated with women’s hormonal status. In further analyses, we included only the cohort of patients over 50 years, with 26 controls (11 men, 15 women) and 95 patients with AS (45 men, 50 women), whose clinical and demographic characteristics are shown in Table 1.

Among patients over 50 years of age (Figure 6C,D), the LV exhibited lower levels of miR-29b in men with AS than control men. Both control women and women with AS showed significantly lower LV expression of miR-29b than their male counterparts. The circulating levels of miR-29b (Figure 6C,D) were significantly elevated in women with AS compared with control women. However, control men and men with AS did not exhibit significant differences in the circulating levels of miR-29b.

**Figure 7:**
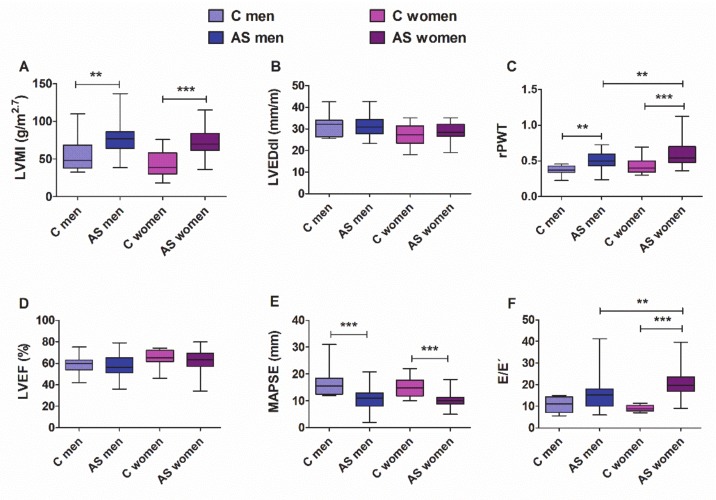
Morphological and functional echocardiographic parameters in controls and men and women with AS who were older than 50 years of age. (**A**) LVMI, LV mass index; (**B**) LVEDdI, LV end-diastolic diameter index; (**C**) rPWT, relative posterior wall thickness; (**D**) LVEF, LV ejection fraction; (**E**) MAPSE, mitral annular plane systolic excursion; (**F**) E/e′, the ratio of peak early transmitral flow velocity to peak early myocardial tissue velocity (e′). Whiskers represent the 10th–90th percentiles. ** *p* < 0.01, *** *p* < 0.001 (two-way ANOVA followed by Bonferroni post hoc test). Control patients: men = 11, women = 15; AS patients: men = 45, women = 50. For MAPSE, control: men = 8, women = 10; AS patients: men = 42, women = 45. For E/e’, control: men = 5, women = 5; AS patients: men = 24 and women = 38.

The morphological and functional echocardiographic features of patients over 50 years of age are depicted in Figure 7. Compared with the controls, both male and female patients with AS showed significantly elevated LVMI and rPWT (Figure 7A,C), and decreased systolic strain in the long-axis (MAPSE, Figure 7E). Both men and women showed preserved LVEF. The LV filling pressures, as reflected by the ratio E/e′ (Figure 7F), increased significantly only in women with AS. When we evaluated differences between sexes, women with AS exhibited higher values of rPWT and E/e′ than men with AS, thus suggesting that women develop hypertrophy with a more concentric geometry and have greater deterioration of diastolic function than men.

**Figure 8:**
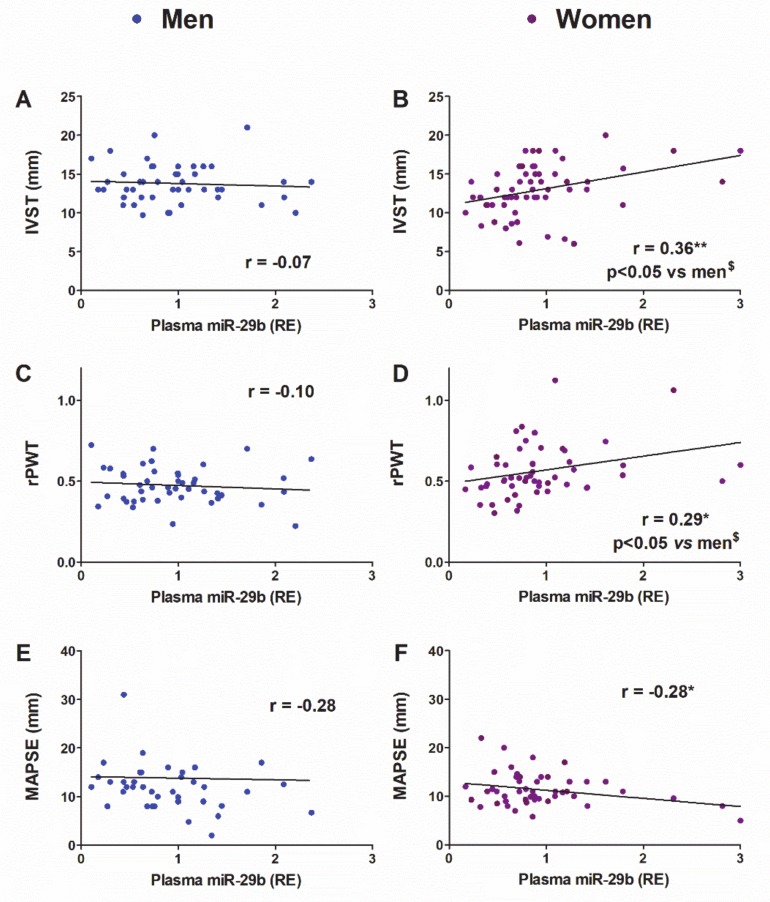
Linear regression and Pearson’s correlation analyses showing the relationship between plasma miR-29b expression and morpho-functional parameters in patients older than 50 years. IVST, interventricular septum thickness in men (**A**) and women (**B**); rPWT, relative posterior wall thickness in men (**C**) and women (**D**); MAPSE, mitral annular plane systolic excursion in men (**E**) and women (**F**). r, Pearson’s correlation coefficient. * *p* < 0.05, ** *p* < 0.01. **^$^**Comparative analysis of women vs. men correlations with cocor.

Our next objective was assessing whether and to what extent the changes in the expression of miR-29b on the LV and/or plasma might explain the pattern of LV morpho-functional remodeling under pressure overload in women and men. As shown in Figure 8, the plasma levels of miR-29b correlated positively with the septal thickness (IVST) and the LV concentricity (rPWT) but correlated negatively with the mitral annular plane systolic excursion (MAPSE) only in women. We further assessed whether the association between miR-29b levels and the parameters studied differed by sex. We confirmed by cocor analysis [37] that the r values of the correlations between plasma miR-29b and LV mass and concentricity differed significantly between women and men (Figure 8).

The expression levels of miR-29b in the LV did not correlate with any echocardiographic parameter in women or men (data not shown).

Given the significant relationships between miR-29b and hypertrophy-related parameters in both TAC-female mice and women with AS, we further assessed whether the preoperative levels of circulating miR-29b might serve as biomarkers to estimate the potential for LV mass normalization (LVMI <51 g/m^2.7^) in patients with AS 1 year after AVR, as we previously demonstrated for miR-133a [7]. In our cohort of patients with AS, the proportion of individuals with normalized LVMI (determined by echocardiography) 1 year after aortic valve replacement was similar in both sexes (16 out of 45 men, 22 out of 50 women; χ^2^, NS).

In a further step, we sought to identify predictors of postoperative LV mass normalization by logistic regression analysis, including as independent variables the preoperative plasma levels of miR-29b, either alone or in conjunction with clinical parameters (body mass index, LV hypertrophy) whose predictive value has been established [7]. The accuracy of the logistic models was determined via the ROC curve.

In women with AS, plasma miR-29b, BMI and PWT, analyzed individually, were significant independent negative predictors of LVMI normalization (Table 2, women with AS, models #1–3). Of note, the inclusion of plasma miR-29b combined with any of the other parameters substantially improved the prognostic accuracy of the model (Table 2, women with AS, models #4 and #5). The model obtained when BMI and PWT were combined with circulating miR-29b (Table 2, model #6 and Table 3) yielded the highest area under the ROC curve (AUC: 0.9 (95% CI: 0.76 to 0.98), *p* < 0.05), with a sensitivity of 81.0% and a specificity of 79.2% (Hosmer-Lemeshow test: χ^2^: 3.14; significance 0.87).

In men with AS, the only independent variable that discriminated patients with normalized LVMI was the BMI (Table 2, men with AS, model #1). Circulating miR-29b lacked predictive power and did not improve the accuracy of the model when combined with the other independent variables (Table 2, men with AS, models #2–6).

## 4. Discussion

The present study provides the first reported evidence of sex-related differences in the regulation of miR-29b in the mouse LV myocardium under pressure overload: miR-29b underwent up-regulation in TAC-females but down-regulation in TAC-males. This dimorphic regulation resulted in opposite consequences on miR-29b target mRNAs, thus resulting in a pattern of LV remodeling that was more fibrotic in males and more hypertrophic in females. From a functional viewpoint, miR-29b appeared to be detrimental to the LV under pressure overload in females but protective in males. In a clinical setting, miR-29b expression in controls and patients with AS reproduced some of the sexually dimorphic features observed in mice. In women with AS, the preoperative expression of miR-29b in plasma was found to be a significant negative independent predictor of LVMI normalization after aortic valve replacement, and it may have potential value as a prognostic biomarker.

During degenerative aortic valve stenosis, the restriction of blood flow through the diseased valve exposes the LV to chronic pressure overload, thereby leading to a remodeling response from the myocardium. Cardiomyocyte hypertrophy and myocardial fibrosis are crucial features of this initially adaptive plasticity that progressively impairs both contractility and relaxation and determines overtime the detrimental evolution toward heart failure [6].

Despite broad sex-related differences in many cardiovascular diseases, including AS [27,28,29,30,31,32,33], biomedical investigations often ignore sex as an essential biological variable, and women and female animals remain under-represented in all stages of cardiovascular research [42].

Most studies investigating sex differences in LV remodeling under pressure overload have been performed in mice subjected to TAC,a well-validated model that mimics aortic valve stenosis [38]. The conventional Rockman procedure uses a single size reference cannula (typically 27 G = 0.41 mm OD) to gauge the constriction in all mice, regardless of sex, bodyweight, or aortic size [30,38,43]. Therefore, this technique produces an identical minimal luminal aortic area in mice with different somatometric characteristics, as occurs between males and females. Hence, the larger the animal, the greater the pressure overload that supports its LV after “standardized” TAC. We must conclude that in such studies the hemodynamic stress is higher in males than in females, thus introducing a confounding factor in the analysis.

In contrast, the modification of the conventional Rockman technique used herein [34] allows for accurate customization of the constriction to the diameter of the aortic arch; consequently, our TAC-males and TAC-females showed identical trans-coarctation gradients even though their body weight difference exceeded 30%.

### 4.1. miR-29b and Myocardial Fibrosis

miR-29b has been found to play a role in pathological myocardial remodeling under pressure overload, but its prognostic effects—beneficial or deleterious—and the cellular hierarchy (cardiomyocytes vs. fibroblasts) that controls its function are unclear [14,26].

Many studies support the canonical view that down-regulation of the miR-29a,b,c family plays a crucial role in the development of persistent fibrosis of various organs, including the heart, through de-repression of ECM-related mRNA targets, particularly in fibroblasts [11,12,13,14,15,16,17,18,19,20,21,22,23,24,25]. Accordingly, miR-29 mimicry may have therapeutic potential in protecting against pathological tissue fibrosis [11,16,21,22,24,44].

Our preclinical results showed that, under baseline load, the expression levels of miR-29b in the LV myocardium were significantly higher in sham-females than in sham-males. Unexpectedly, after 4 weeks of identical hemodynamic stress, miR-29b exhibited opposite regulation trends in TAC-males (down-regulation) and TAC-females (up-regulation). These sex-related differences in miR-29b expression and regulation were prevented in OVX females. However, the castration of males did not modify the expression of miR-29b. These results suggest that gonadal estrogens may be transcriptional activators of miR-29b in the LV in young females under both physiological and pathological load conditions. Accordingly, estradiol-induced the expression of miR-29b in cardiac fibroblasts in females but not in males. These results are consistent with in silico studies showing the presence of putative estrogen response elements in the promoter regions of miR-29 clusters [45].

Our results are also in line with the sexually dimorphic, estrogen-dependent, hepatic regulation of miR-29a,b reported in mice. Specifically, down-regulation of miR-29a,b in males correlates with the early development of liver fibrosis after treatment with CCl4. In contrast, estrogens protect females against CCl4-induced down-regulation of miR-29a,b and subsequent fibrosis [21].

Experimental studies based on in vivo models of stress-induced organ fibrosis and cultured fibroblasts under fibrotic stimuli [12,13,14,17,18,19,20,24,25] indicate that down-regulation of mir-29b participates in the pro-fibrotic effects of TGF-β1, the master cytokine involved in LV remodeling under pressure overload [29,35,39]. Our results in male mice subjected to TAC and in male cardiac fibroblasts under TGF-β1 stimulation support the contribution of miR-29b down-regulation and subsequent post-transcriptional de-repression of its targets, such as Collagens I and III, to the fibrogenic effect of TGF-β1. In contrast, in female TAC-mice and LV female fibroblasts, collagen overexpression was significantly lower than that in males and did not involve transcriptional repression of miR-29b by TGF-β1. These results strongly suggest sex-related differences in the molecular signaling mechanisms that are activated by TGF-β1 and trigger myocardial fibrosis. In support of this possibility, our previous findings in patients with AS [29] demonstrate that, in the LV, expression of genes encoding fibrosis-related markers (Col1α1, Col3α1, and fibronectin) directly correlates with the expression of Smad-2 (a major effector of TGF-β1 signaling) in men with AS but not in women with AS. Furthermore, in vitro studies in isolated cardiac fibroblasts from rats have shown that estradiol induces Col1α1 and Col3α1 transcript up-regulation in males but down-regulation in females [46].

In summary, our findings show sex-differential regulation of the signaling mechanisms triggered by pressure overload to induce LV fibrosis. In males, TGF-β induces miR-29b down-regulation and subsequent de-repression of its mRNA targets encoding fibrillar collagens, thus contributing to the development of myocardial fibrosis. This fibrogenic mechanism does not appear to be operational in females under identical hemodynamic stress. This aspect, together with the estrogen-dependent transcriptional activation of miR-29, renders females more resistant to fibrosis development, as indicated by the lower expression of fibrosis markers in female mice relative to males.

Overall, these results are in line with findings from previous studies showing that women with AS, compared with men, exhibit lower expression of mRNAs encoding collagens I and III [46] and more severe LV interstitial fibrosis [47]. Indeed, we do not exclude the contribution of other hormonal and non-hormonal, sex-intrinsic, and/or epigenetic mechanisms to the sexually dimorphic LV remodeling, as evidenced by the differences exhibited by cardiac fibroblasts cultured in the absence of sex steroids (present results) and postmenopausal women with AS (current results and 29,46,47).

### 4.2. miR-29b and Cardiac Hypertrophy

Whether miR-29b regulates hypertrophic processes in the heart has scarcely been investigated. A recent report, [26] using a loss-of-function approach has found that miR-29a,b,c deficiency protects TAC-male mice against pressure overload-induced LV hypertrophy and fibrosis. The authors postulate a dominant cardiomyocyte pro-hypertrophy effect of the miR-29 family members, which subsequently promotes a fibrotic response from fibroblasts. However, this process incongruously occurs with a parallel down-regulation of miR-29b in the LV from both wild type TAC-mice and patients with AS. Therefore, this pro-hypertrophic response might be due to the dominant effect of miR-29a on cardiomyocytes [48].

Herein, echocardiographic follow-up of mice revealed that the LV hypertrophy developed to overcome identical hemodynamic stress was more pronounced in TAC-females than in TAC-males. Consistently with our findings, the sarcomeric protein β-MHC, a classic marker of pathological cardiac hypertrophy in rodents [40], was up-regulated to higher levels in the LV in TAC-females.

An exciting finding in female mice was the direct relationship between the LV expression levels of miR-29b and echocardiographic parameters of hypertrophy (IVS, PWT, and LVM). Hence, female mice with the highest LV expression of miR-29b displayed the most severe cardiac hypertrophy, thus supporting a hypertrophic effect of miR-29 [26]. However, this relationship followed the opposite trend in males, thus suggesting a protective effect of miR-29b against cardiomyocyte hypertrophy. Similarly, cardiomyocytes derived from mouse embryonic stem cells undergo atrophy when cultured in the miR-29b conditioned medium [14].

We provided further insights into this dimorphic behavior of miR-29b by demonstrating regulation of its target GSK-3β, whose repression is associated with the pro-hypertrophic effects of miR-29 in pressure overloaded LV [26]. GSK-3β expression was significantly reduced by TAC in the LV in females but up-regulated in the LV in TAC-males. Therefore, miR-29b up-regulation, through GSK-3β repression, is associated with the more severe hypertrophy developed in females than males under pressure overload.

### 4.3. Correlation between LV miR-29b Expression and Morpho-Functional Remodeling

An important finding of our study was the strong influence of the sex-biased regulation of miR-29b on the morphological, geometric and functional remodeling of the LV in both sexes in TAC-mice. Compared with TAC-males, TAC-females exhibited more severe hypertrophy with more concentric geometry, a greater reduction of systolic function in both the short-axis (LVEF) and long-axis (MAPSE), as well as higher filling pressures (E/e’). The sexual differences that we observed in mice are fairly consistent with those exhibited by patients with AS (concentricity and diastolic dysfunction present results and [31,49]), thus supporting the relevance of the preclinical results. Still, cardiac remodeling in clinical settings is influenced by uncontrolled variables such as patient comorbidities (such as diabetes, obesity, coronary artery disease or hypertension), the degree of valve stenosis or an undetermined duration of hemodynamic stress. 

An intriguing finding in our study is the opposite relationship between miR-29b levels and the echocardiographic morpho-functional parameters exhibited by males and females. In female mice, the LV levels of miR-29b correlated positively with hypertrophy, concentricity of the remodeling and diastolic dysfunction (E/e’), and negatively with systolic function in the long and short axes (MAPSE and LVEF). In males, higher the LV levels of miR-29b were associated with less severe hypertrophy, concentricity, and systolic and diastolic dysfunction. These results suggest that, from a remodeling perspective, miR-29b is detrimental to the myocardium under pressure overload in females but is protective in males.

In females, the remodeling of the sarcomeric cytoskeleton in cardiomyocytes under hemodynamic load might be responsible for greater stiffness and lower LV compliance, thus resulting in more severe impairment of the cardiac function [50] than that in males. Additionally, aberrant collagen cross-linking has been reported to be more detrimental to LV diastolic and systolic performance than total collagen deposition in the ECM [4,51].

### 4.4. miR-29b Regulation in Patients with Aortic Valve Stenosis

The miR-29b transcriptional sex differences observed in mice, both basally and after TAC, suggested that its participation in sexual dimorphism phenomena in clinical settings is plausible and might explain some of the discrepancies in published data. The experimental data also showed that hormonal status is relevant for females; therefore, we assessed whether female patients, with or without pressure overload, would show recapitulation of this behavior.

In control women (aged 28–83 years), age and miR-29b levels in either LV or plasma correlated negatively. However, such a relationship was absent if the younger than 50 years were excluded from the analysis. Moreover, control post-menopausal women exhibited significantly lower expression levels of miR-29b than their younger counterparts. These results strongly suggest an influence of hormonal status in miR-29b expression, as we observed in female mice. Otherwise, among patients over 50 years of age, a usual age range for patients with degenerative AS, both control men and AS men showed higher miR-29b myocardial values than their female counterparts. This finding supports that, in addition to the hormonal status of women, intrinsic, non-hormonal factors throughout life also contribute significantly to sex differences in LV remodeling [30,31].

To circumvent the influence of the hormonal status of women, subsequent analyses only included patients older than 50 years. Men with AS, like TAC-male mice, exhibited LV down-regulation of miR-29b. However, their circulating levels remained unaltered. In postmenopausal women with AS, like in OVX-TAC-mice, miR-29b was not regulated in the LV. In contrast, the plasma levels of miR-29b were significantly elevated in women with AS compared with their control counterparts.

The discrepancy between LV and plasma miR-29b expression in women might have been due to the confounding effect of other systemic sources of miR-29b in the circulation. Aside from the heart, other sources can contribute to circulating miR-29b and, therefore to cardiac remodeling. In this regard, platelets, monocyte/macrophages, and endothelial cells are highly enriched in microvesicles containing miR-29 [52,53]. Along with myocardial resident cells, infiltrating immune cells, vascular endothelial cells and platelets are major culprits in the pathological remodeling of the myocardium after hemodynamic stressors (i.e., pressure or volume overload), metabolic diseases (obesity, diabetes), injuries (myocardial infarction), etc. [54].

The potential value of circulating miRNAs as biomarkers for diagnostic, prognostic, and therapeutic stratification purposes is a matter of growing interest in clinical medicine, particularly in cardiovascular diseases [55]. Therefore, we further assessed whether the sex-related differential regulation of miR-29b is related to differences in cardiac morphology or function. Our cohort of individuals over 50 years of age exhibited sex-related differences somewhat consistent with findings from other clinical studies [27,31,49] and in TAC-mice. Compared with controls, patients of both sexes with AS showed significant and equivalent hypertrophy and deteriorated systolic function in the long-axis (MAPSE). Both men and women with AS exhibited preserved LVEF. Men and women with AS switched to a more concentric LV chamber than that observed in controls, but women did so to a greater extent than men. Only women with AS showed higher LV filling pressure (E/e’) than that observed in control women and men with AS, indicating a more severe deterioration of the diastolic function.

Our group previously underscored the value of preoperative plasma levels of remodeling-related miRNAs as prognostic biomarkers of reverse remodeling after aortic valve replacement in patients with AS [7,56]. An important finding of the present study was the positive relationship of preoperative miR-29b plasma levels with hypertrophy (IVST) and concentricity (rPWT) only in women with AS. These relationships did not hold true for the expression of miR-29b in the LV, thus further suggesting a significant role of non-myocardial miR-29b sources in myocardial remodeling under pressure overload in women. This feature was female sex-specific and unrelated to the women’s hormonal status because all patients in the cohort were older than 50 years. In line with our results, the cardiac hypertrophy developed by hypertensive patients with target-organ damage has been shown to have a positive relationship with the plasma levels of miR-29b [57].

Furthermore, we attempted to ascertain whether the preoperative circulating miR-29b levels, together with some other validated clinical and echocardiographic parameters, could predict the potential for LV mass normalization 1 year after aortic valve replacement. We found that this was the case for women with AS, in whom miR-29b levels increased the predictive power of regression equations in forecasting mass normalization 1 year after AVR. This finding is meaningful, given that both the severity of preoperative LV hypertrophy and the persistence of hypertrophy after aortic valve replacement are risk factors for poor long term outcomes including heart failure and death [58,59]. Furthermore, the pace at which reverse remodeling occurs early after TAVI (percutaneous transcatheter aortic valve implantation) has a prognostic influence on hospital readmissions, heart failure, circulating BNP levels, and probably the quality of life [60].

The present study supports the notion that circulating miR-29b may contribute to establishing individualized risk profiles of female patients with AS before surgery, and providing information on the potential for regression of LV myocardial remodeling after aortic valve replacement. This information may prompt more proactive therapeutic strategies after surgery in patients with less favorable profiles of reverse remodeling and may even help to conform important clinical decisions, such as the mode and timing of treatment, according to the specific regression potential of the patient [61].

miR-29b is an important factor in the myocardial adaptation to pressure overload that functions in a sex-dependent manner. Its therapeutic modulation may be a favorable strategy but cannot be performed indiscriminately. A corollary to our work is that in alternative therapeutic avenues of basic and clinical research, such as the use of miR modulators [12,26], the eventual sexually dimorphic responses that these molecules can elicit should be considered. Ignoring this fact may result in the achievement of the clinical improvement sought, blunted efficacy or detrimental consequences depending on the sex of the patient.

## Figures and Tables

**Table 1:** Clinical and demographic characteristics of control and aortic stenosis patients older than 50 years.

	Men > 50 Years		Women > 50 Years	
	Control *n* = 11	AS *n* = 45	Control *n* = 15	AS *n* = 50
Hyperlipidaemia	33.3%	32.7%	13.3%	42.3% *
Obesity	54.5%	31.1%	26.7%	44.2%
Hypertension	72.7%	71%	53%	77%
Diabetes mellitus	9.1%	31.2%	6.6%	13.5% ^#^
Age (mean ± SD)	59.8 ± 6.5	69.3 ± 9.0	61.6 ± 9.3	72.5 ± 7.2

AS, aortic stenosis; SD, standard deviation, *χ*^2^: * *p* < 0.05, control vs. AS; # *p* < 0.05, women vs. men.

**Table 2:** Logistic regression analysis predictive models of LV mass normalization one year after aortic valve replacement in aortic stenosis (AS) patients.

	Model	Plasma miR-29b OR (95% CI)	BMI OR (95% CI)	PWT (mm) OR (95% CI)	AUC
**AS women**	1	0.12 (0.02–0.75)			0.73
	2		0.81 (0.69–0.95)		0.71
	3			0.62 (0.44–0.89)	0.72
	4	0.14 (0.02–0.88)	0.80 (0.66–0.95)		0.83
	5	0.14 (0.22–0.9)		0.56 (0.33–0.93)	0.82
	6	0.16 (0.03–0.93)	0.81 (0.66–1.0)	0.63 (0.4–1.0)	0.9
**AS men**	1	0.93 (0.35–2.47)			0.53
	2		0.81 (0.67–0.99)		0.7
	3			0.86 (0.62–1.18)	0.55
	4	0.75 (0.27–2.1)	0.81 (0.65–0.99)		0.72
	5	0.97 (0.36–2.61)		0.83 (0.58–1.2)	0.57
	6	0.78 (0.27–2.24)	0.81 (0.66–1.0)	0.85 (0.58–1.25)	0.74

The models include the independent variables circulating miR-29b in plasma, body mass index (BMI), and posterior wall thickness (PWT). The variables were analyzed individually (models 1 to 3) and combined (models 4 to 6). AUC, area under the ROC curve; CI, confidence intervals; OR, odds ratio. (Men *n* = 38, women *n* = 46).

**Table 3:** Logistic regression predictive model of LVM normalization one year after aortic valve replacement in AS women (#6).

Model #6	Variable	OR (95%CI)	B	p
AS women	Plasma miR-29b	0.16 (0.03–0.93)	−1.83	0.041
	BMI (1 unit)	0.81 (0.66–1.0)	−0.21	0.037
	PWT (mm)	0.63 (0.4–1.0)	−0.46	0.044

B, unstandardized coefficient; BMI, body mass index; CI, confidence intervals; OR, odds ratio; posterior wall thickness (PWT) (Men *n* = 38, women *n* = 46).

## Supplementary Materials

The following are available online at https://www.mdpi.com/2073-4409/9/4/833/s1, Figure S1: Mean trans-coarctation gradients determined by Doppler echocardiography 4 weeks after transverse aortic constriction (TAC); Figure S2: Percent change of morphological and functional echocardiographic parameters in male and female mice 4 weeks after TAC versus basal condition; Figure S3: Linear regression and Pearson’s correlation analyses between miR-29b expression in the LV and plasma with age in control patients of any age.
