# Supplementary material for: Sex-Specific Regulation of miR-29b in the Myocardium Under Pressure Overload is Associated with Differential Molecular, Structural and Functional Remodeling Patterns in Mice and Patients with Aortic Stenosis
Source: Cells. 2020 Mar 30;9(4):833. doi: 10.3390/cells9040833 (PMC7226763; doi:10.3390/cells9040833)
Supplement: Supplementary file 1 [file cells-09-00833-s001.pdf]

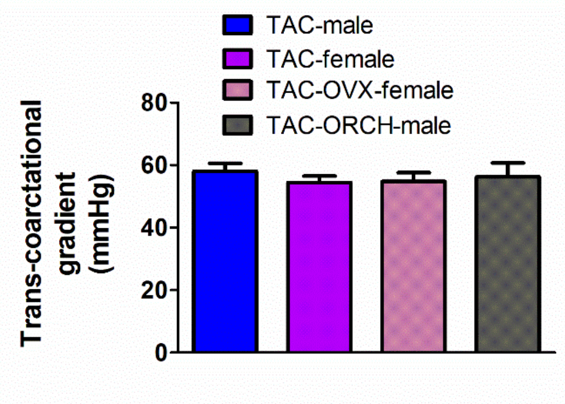

Figure S1. Mean trans-coarctational gradients determined by Doppler echocardiography 4 weeks after transverse aortic constriction (TAC). Male (n = 18), female (n = 29), ovariectomised (OVX) (n = 5) and orchiectomised (ORCH) male (n = 5) mice.

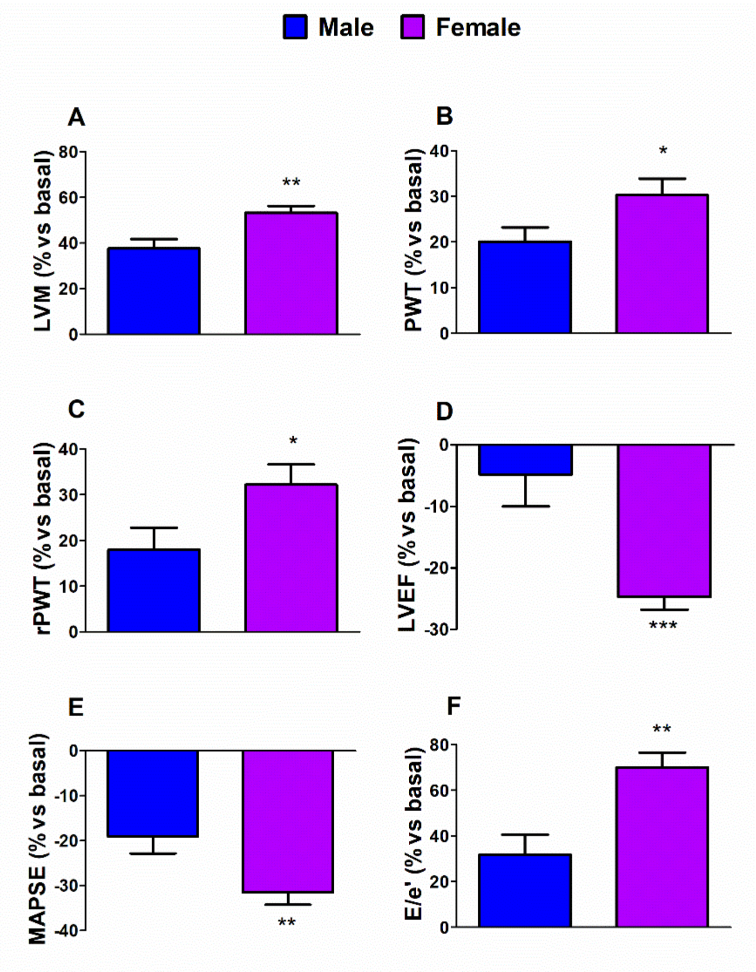

Figure S2. Percent change 4 weeks after TAC versus basal of morphological and functional echocardiographic parameters in male and female mice. A: LVM, LV mass; B: PWT, posterior wall thickness;

C: rPWT, relative posterior wall thickness; D: LVEF, LV ejection fraction; E: MAPSE, mitral annular plane systolic excursion; F: E/e′, ratio of peak early transmitral flow velocity to peak early myocardial tissue 1 velocity (e′). Data are means ± SEM. *p < 0.05, **p < 0.01, ***p < 0.001 (Unpaired t-test).

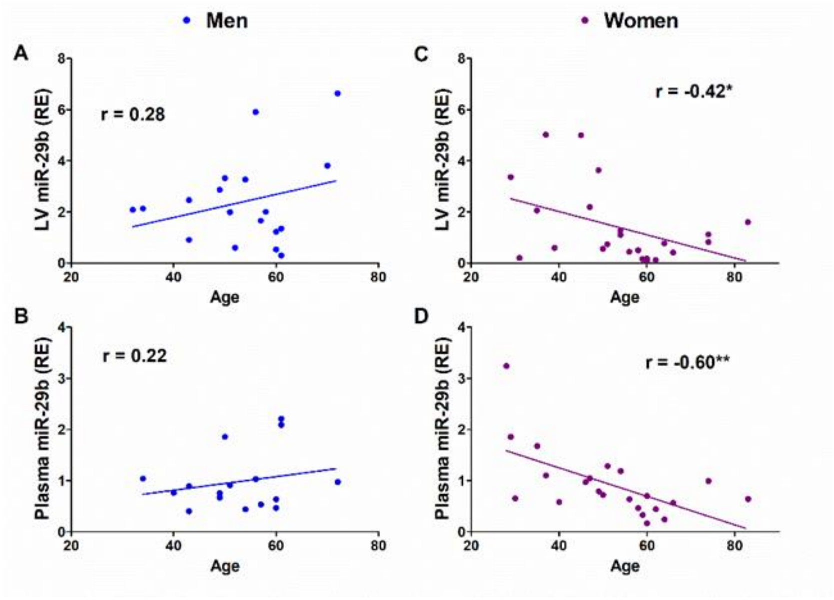

Figure S3. Linear regression and Pearson's correlation analyses between miR-29b expression in the LV and plasma with age in control patients of any age. A: miR-29b relative expression in the LV (RE vs RNU6b) and B: plasma (RE vs Cel-39) from control men. C: miR-29b relative expression in the LV (RE vs RNU6b) and D: plasma (RE vs Cel-39) from control women. r, Pearson's correlation coefficient (*p < 0.05, **p < 0.01).
